# The Leprosy Scare

**Published:** 1888-01

**Authors:** 


### *THE LEPROSY SCARE.*

It will be remembered that, some years ago, it was proposed to bring from the Pacific coast two persons afflicted with leprosy; the intention being to turn their misfortune into a means of making money, by the public exhibition of their affliction. Much popular alarm was caused by the announcement, and an intimation that the authorities would not permit such an exhibition, led to the abandonment of the project. It would be to the public good if all such exhibitions of personal deformities and diseases could be summarily suppressed.

The incident showed, however, how great a dread there exists, in the popular mind, of the disease, and this has been more strongly indicated by the excitement in Philadelphia, due to the presence in that city of two cases of leprosy, whereby the community was thrown into a state of terror.

It appears that, about a year ago, a woman and her daughter came to this country from Brazil, locating in Philadelphia. The woman consulted Dr. Van Harlingen concerning a "Skin Disease", which the doctor at once recognized as a case of leprosy. A few months ago, he read a paper at the County Medical Society, in which these cases were described, and one patient was shown to the members.

The fact that there was a leper in the city then, soon reached the ears of the ubiquitous reporter, and was worked up into a sensation in short order. The Board of Health took cognizance of the matter, and demanded from Dr. Van Harlingen the address of the unfortunates, which the doctor refused to give, as involving a breach of professional honor. The Board then added the disease to the list of contagious affections, of which, under penalty, notice must be given to the authorities, and on Dr. Van Harlingen's continued refusal, fined him $100 for violation of the law. The upshot was, that the patients voluntarily reported to the health office, and were immediately isolated at the Municipal Hospital for contagious diseases.

A number of prominent physicians of Philadelphia, feeling that the doctor had been hardly treated, united in paying his fine, and their letter rather reflects upon the action of the Board.

The popular conception of the awful danger of contagion undoubtedly derived from the sacred writings, but there is no positive evidence that the leprosy of the Bible is the disease that is now designated by that name. That true leprosy is contagious in the sense, that there is absolute danger to a community from the existence of a leper in its midst, has never been proved. In London, there is almost always at least a dozen cases coming from India and other southern colonies of the Empire. Though they are permitted perfect liberty, there is not on record a single case of the disease which had its origin from contact with the diseased individual in England. Liveing—perhaps the most eminent dermatologist in England—asserted to the writer, that he had known lepers coming to England, in the early stages of the disease, entirely recover. The Royal College of Physicians, in answer to some expressed fears of danger from the presence of the disease in so crowded a centre of population, appointed a committee to investigate, and the text of their report is as follows:

"The committee are of opinion that if there is any elements of contagion in leprosy, they are not more to be dreaded than are those in the case of syphilis, which is not commonly considered to justify segregation on the part of those affected, and that they be lieve that leprosy is not contagious in the conventional sense of the term, but, if at all, it is only so in a low degree, and under exceptional circumstances."

We are of the opinion, therefore, that Dr. Van Harlingen's action in the matter was entirely justifiable, and that the outcry against him, on the part of the newspapers, was in no way warranted by the facts. In the words of an editorial in the *Medical and Surgical Reporter*, "we feel assured that others who will study the subject carefully will conclude that nothing is to be feared, in England or North America, from the presence of a few lepers. They are to be treated, as other sick folk, with kindness and the best skill available, keeping in view the remote possibility that the disease may spread from them. But, until a single authentic instance of its communication by contagion in this country can be adduced, it is the height of folly to demand their separation from their fellows, to treat them as if they had small-pox or scarlet fever, and to excite the laity to an unreasonable fear and horror of them. Physicians who entertain other opinions in regard to the contagiousness of leprosy, may effect all that is necessary for the protection of the public without exaggeration of its danger. At present, the need seems to be that exaggerations which have been set in motion should be checked, and the confidence of the people in the ordinary methods of medical and sanitary protection should be re-established."

